# Factors related to the internal social capital of elderly-caring social organizations: a cross-sectional study in Chongqing, China

**DOI:** 10.1186/s12913-023-09912-8

**Published:** 2023-08-23

**Authors:** Fuqin Xu, Shuo Ding, Guoqing Liu, Zhengsheng Wang, Benjamin Otsen, Kai Ji, Xin Zheng, Ren Chen

**Affiliations:** 1 School of Health Services Management, Anhui Medical University, Hefei, 230032 China; 2 Directorate of Research, Innovation and Consultancy, University of Cape Coast, Private Mail Bag, Cape Coast, Ghana; 3 Key Laboratory of Public Health Social Governance, Philosophy and Social Sciences of Anhui Province, Hefei, Anhui 230000 China

**Keywords:** Social organization, Aging, Internal social capital, Influencing factors

## Abstract

**Background:**

Aging has given birth to the demand for high-quality elderly care service and social organizations (ESOs) are gradually taking on a supportive role in the field of elderly care services.. In view of this, our study is designed to examine influencing factors of social capital within the elderly-caring social organizations.

**Method:**

The study was conducted in four districts of Chongqing Province and a multi-stage random sampling method was used to sample 80 ESOs as subjects for the research. Through a meticulously crafted questionnaire, we gathered valuable data on internal social capital, basic information about the organization, and other variables. Univariate and Binary Logistic Regression analysis were performed on the data to explore the factors associated with social capital within the elderly-caring social organizations using IBM SPSS version 26.0.

**Result:**

The results showed that 67(83.8%) OF ESOs surveyed were A-type institutions and 13(16.3%) B-type institutions. Among them, 49(61.3%) institutions covered an area of more than 50m^2^.The institutions of ≤ 2 years were 33(41.3%), 21(26.3%) were established for ≤ 3 ≤ 8 years, and residual 26(32.5%) were institutions ˃ 8 years old. ESOs that possessed 4–6 management employees were 52.422 times more likely to score high for social support (*p*<0.05, OR = 52.422). Accommodating special care objects and hiring 16–30 employees were significantly linked to the shared language and shared vision dimension (*p*<0.001, OR = 0.8) and (*p*<0.05, OR = 8.672), respectively. and the overall social capital dimension (*p*<0.01, OR = 0.221) (*p*<0.05, OR = 5.730).

**Conclusion:**

ESOs with different basic conditions have different amounts of social capital. Factors such as the presence of special care and volunteer staff, a certain number of full-time staff as well as types of services rendered are accompanied with the higher internal social capital of ESOs.

**Supplementary Information:**

The online version contains supplementary material available at 10.1186/s12913-023-09912-8.

## Introduction

In 2000, China formally entered an aging society. The seventh census bulletin of the National Bureau of Statistics showed that the population over 60 years old was about 260 million people, accounting for 18.7% of the total population of China. Of this figure the population over 65 years old accounted for 13.5%., [1]. This set of data illustrates the fundamental characteristics of China's aging society, namely, rapid pace, large scale, and the phenomenon of aging before becoming affluent. To proactively address the aging phenomenon, the 14th Five-Year Plan for the Development of the National Aging Cause and the Elderly Care Service System proposed the need to expand efficient and high-quality elderly care services. This has consequently urged all kinds of ESOs to increase the supply of elderly care services while improving the quality of elderly care services and promoting the high-quality development of the elderly care service industry [2, 3].

The participation of social organizations in the elderly care services has become an important supplement to the elderly care system in China, bridging the shortcomings of public elderly-care and home-based care in providing adequate and high-quality elderly care services [4]. De Montigny JG pointed out in a study that multi-sector collaboration, specifically the cooperation between the government and social organizations, can enhance the quality and efficiency of social services to a certain extent, especially in the field of healthcare for older adults [5, 6]. Previous studies had shown that organizations and communities with higher social capital were more efficient, creative and could provide better services than organizations with lower social capital [7]. This meant that the research on ESOs from the perspective of enhancing internal social capital has a certain practical value.

As a developing concept, Social Capital theory can be traced to Bourdieu 's study of social space. Coleman and Putnam promoted the development of the concept of social capital [8, 9]. Putnam believed that social networks and social norms were very important for social cooperation, and social networks and social support formed the core elements of internal social capital [10, 11]. Internal social capital is a multidimensional concept. It is composed of social network, social trust, social support, social norms, common language and common vision [12]. The great potential of social organizations to participate in old-age care has not yet been fully realized, and therefore research on ESOs from the perspective of internal social capital provides new ideas and paths for stimulating the potential of institutional elderly care.

Literature on internal social capital were the basis of this research. Considering that organizational performance is an intuitive indicator of organizational ability, scholars have found that internal social capital has a positive and significant impact on organizational performance, especially on non-financial performance, which included the services provided by institutions [13–15]. Currently, the majority of research focuses on the relationship between internal social capital and organizational performance, while mentioning little about the factors influencing internal social capital. Based on this, our study sought to explore the subject of internal social capital theory; which includes its five dimensions: social network, social trust, social support, social norms, common language and common vision. The objective of this study is to identify key factors influencing the internal social capital of elderly-caring social organizations and to take targeted measures to enhance the stock of internal social capital, thereby improving the elderly care environment and optimizing the elderly care industry.

## Materials and methods

### Study design and data collection

This study was approved by the Biomedical Ethics Committee of Anhui Medical University (No. 20180181) and was officially launched in Chongqing from July to August 2022.

Chongqing is located in the southwestern part of China. In 2021, the population aged 65 and above in Chongqing was 5.70 million, accounting for 17.75% of the total population. This proportion is still on the rise, indicating a continuous deepening of population aging in the region [16]. In 2022, the Chongqing Municipal People 's Government issued the ' 14th Five-Year Plan for the Construction of Chongqing 's Pension Service System (2021–2025) ' to accelerate the construction of a multi-level pension service system comprising home-based care, community-based care, fully developed institutions, and medical care. First of all, we used a multi-stage stratified random sampling method. In the first stage, random sampling was conducted in 26 districts of Chongqing, and four districts (D, J, T and R) were selected as survey points. In the second stage, we randomly sampled 80 organizations from the list of ESOs provided by the local civil affairs bureau. At the third stage, we randomly selected a superintendent of an institution to conduct a more precise investigation of the organization's basic situation, thereby enhancing the accuracy of the study. The superintendent includes president, director, head, curator, and other personnel, who had a thorough understanding of the organization. The investigators are comprised of graduate students from Anhui Medical University and Chongqing Medical University. Regarding the questionnaire content, our investigators underwent professional training prior to commencing the survey. This training aimed to enhance their comprehension of the questionnaire, ensuring a smoother and more efficient survey process. The staff from the local Civil Affairs Bureau guided the research team to administer the questionnaire to the superintendent organization. Preceding this, the investigators introduced the basic content of the questionnaire, ensured that the content of the investigation would not be misunderstood, and obtained the informed consent of the respondents. We had rigorously managed and monitored the data collection and processing process, ensuring that each selected participant actively responded to our survey questionnaire or engaged in relevant data collection. After rigorous screening, all the responses from the selected sample were included in the data analysis.

### Measurement of social capital

In this study, we developed an internal social network scale by combining the social capital assessment tool developed by the World Bank and previous research. At the same time, experts reviewed and approved the content of the scale. The scale aims to reflect the basic characteristics of Elderly Service Organizations (ESOs) in Chongqing [17–20]. The scale is comprised of two sections, focusing on both the organizational basic information and the internal social capital of ESOs. For the measurement of internal social capital, there were five dimensions: social trust, social support, social network, social norms, common language and common vision of which included 26items. In each item, we used the Likert five-point scale to measure and score, where 1 = “completely disagree”,2 = “not agree”,3 = “general”,4 = “partially agree”,5 = “completely agree”. To comprehensively reflect the status of internal social capital of ESOs, We calculate the total score of internal social capital (0–130) by summing up the scores from the five dimensions. In performing the binary logistic regression, we dichotomized the scores of total score and each dimension by taking the median as the cut-off: social support (high [≥ 25] and low [< 25]), social network (high [≥ 24] and low [< 24]), social trust (high [≥ 25] and low [< 25]), shared language and shared vision (high [≥ 25] and low [< 25]), social norm (high [≥ 25] and low [< 25])and total score (high [≥ 120] and low [< 120]). The overall Cronbach’s α coefficient for the scale was 0.915, indicative of good reliability and representativeness.

### Measurement of other variables

The basic situation of the organization we investigated included per capita floor area (≤ 50, > 50), founding time (≤ 2,3–8, ≥ 9 years), full-time staff (≤ 15, 16–30, ≥ 31),management employees (≤ 3, 4–6, ≥ 7), have volunteers (Yes, No), types of registration (1, > 1), operation pattern (public construction and operation, public construction and private operation,private construction and operation), types of services (≤ 3, 4–6, ≥ 7) self-care (Yes, No), total care (Yes, No), special care (Yes, No), home care service for older adults (Yes, No).

### Statistical analysis

To ascertain potential influencing factors, we performed univariate analysis and binary logistic regression using IBM SPSS Statistics 26.0. Since the data did not conform to the properties of normal distribution, we used the composition ratio N (%), the median number and interquartile range to describe the basic composition of the data. Next, different single factor analysis methods were adopted according to whether the independent variables are binary variables. The two-category variables were tested by Mann–Whitney test to obtain the U value, and the three-category variables were tested by Kruskal–Wallis test to obtain the H value.

Studies had shown that a binary logistic model can still be used for analysis with a small sample size [21–23]. Five meaningful factors in the univariate analysis were included in the binary logistic regression (Special care, Volunteer, Types of services, Management employees, full-time employees). According to the median of the five dimensions, they were divided into high and low categories.

## Results

### Descriptive analysis

Fundamental state and the results of univariate analysis of the ESOs are shown in Table 1. We surveyed 80 institutions in Chongqing, which consisted of 67(83.8%) A-type institutions and 13(16.3%) B-type institutions (A-type refers to social organizations engaged in elderly care services that have been filed or registered with civil affairs departments at all levels, providing comprehensive elderly care services. B-type refers to urban community elderly care service centers (stations) and rural township elderly care service centers, which only offer daily activities and dining services.). Among them, 49(61.3%) institutions covered an area of more than 50m^2^.The institutions of ≤ 2 years were 33(41.3%), 21(26.3%) were established for 3–8 years, and residual 26(32.5%) were institutions ≥ 9 years. Most institutions 55(68.8%) had fewer full-time employees, about ≤ 15, and only a few institutions 9(11.3%) had more than 30 employees. For managerial roles, 49(61.3%) institutions had ≤ 3 managers, 20(25.0%) units had4-6 managers, and the remaining 11(13.8%) institutions had more than 6 managers. Additional descriptive analysis showed that on the types of services rendered, 57(71.3%) of the organizations offered more than 7 services, 25.0% offered 4–6, and the remaining provided only ≤ 3. More than half of the subjects 67(83.8%) had not recruited volunteers, and 67(83.75%) provided home care service for older adults and 67(83.8%) registered in various ways. Further analysis showed 22(27.5%) were in the category of public construction and operation type, 26(32.5%) were public construction and private operation, while 32(40.0%) were private construction and operation. The most common type of objects was self-care 79(98.75%), followed by total care 70(87.5%), and lastly by special care 35(43.75%).

**Table 1:** Basic Situation and the results of the univariate analysis of ESOs (*N* = 80)

Variables	N (%)	M(IQR)	Z/H	*P*-value
**Per capita floor area(m**^**2**^**)**				
≦50	31(38.8%)	118(28)	-1.573	0.116
>50	49(61.3%)	121(14)		
**Founding time(years)**				
≤ 2	33(41.3%)	122(17)	4.957	0.084
3–8	21(26.3%)	113(26)		
≥ 9	26(32.5%)	119.5(28)		
**Full-time staff**				
≤ 15	55(68.8%)	118(27)	6.858	**0.032**
16–30	16(20.0%)	122(11)		
≥ 31	9(11.3%)	98(29)		
**Management employees**				
≤ 3	49(61.3%)	118(26)	7.214	**0.027**
4–6	20(25.0%)	123(10)		
≥ 7	11(13.8%)	108(29)		
**Owning Volunteer Staff**				
Yes	13(16.3%)	122(9)	-2.070	**0.038**
No	67(83.8%)	118(26)		
**Types of Registration**				
1	13(16.3%)	118(6)	-0.229	0.819
>1	67(83.8%)	120(28)		
**Operational pattern**				
Public construction and operation	22(27.5%)	117(26)	2.485	0.289
Public construction and private operation	26(32.5%)	118(26)		
Private construction and operation	32(40.0%)	122(14)		
**Types of services**				
≤ 3	3(3.8%)	121(3,3,1^a^)	6.573	**0.037**
4–6	20(25.0%)	123(12)		
≥ 7	57(71.3%)	118(26)		
**Service object type**				
**Self-care**				
Yes	79(98.75%)	120(27)	-0,457	0.648
No	1(1.25%)	(2^a^)		
**Total care**				
Yes	70(87.50%)	119.5(24)	-1.593	0.111
No	10(12.50%)	125.5(12)		
**Special care**				
Yes	35(43.75%)	99(30)	-3.781	<**0.000**
No	45(56.25%)	122(12)		
**Home care service for older adults**				
Yes	13(16.25%)	120(35)	-0.315	0.753
No	67(83.75%)	120(25)		

^a^Because the data is too small to derive M (IQR), the original data is listed

### Univariate analysis

In conducting the univariate analysis, the total score of internal social capital (the sum of the scores of the five dimensions) was held as the dependent variable, with the basic characteristics of the organization as the independent variables. It was observed that only full-time staff, managers, the number of volunteer services and professional care had a statistically significant association with internal social capital. As shown in Table 1.

### Results of the binary logistic regression analysis

To assess the relationship between the statistically significant independent variables from the univariate analysis and the derived dimensions of internal social capital, a binary logistic regression was performed. On the dimension of social trust, an improvement was seen in those institutions with receiving special care objects (*p*<0.001, OR = 0.047). Similarly with social norm, organizations which had special care objects (*p*<0.001, OR = 0.025), rendered 4–6 services (*p*<0.05, OR = 57.953) and hired 16–30 employees (*p*<0.01, OR = 24.247) showed more higher scores for social norms than else. In exploring the relationship between social support and significant variables from the univariate analysis, we found that providing special care (*p*<0.05, OR = 0.203) and the presence of volunteer staff (*p*<0.05, OR = 11.721) positively impacted social support score. ESOs that possessed 4–6 management employees were 52.422 times more likely to score high for social support (*p*<0.05, OR = 52.422). Accommodating special care objects and hiring 16–30 employees were significantly linked to the shared language and shared vision dimension (*p*<0.001, OR = 0.8) and (*p*<0.05, OR = 8.672), respectively. As shown in Table 2.

**Table 2:** Binary logistic regression of social capital and its five dimensions

Variables	Subdomains	Social trust	Social norm	Social support	Social network	Common Language and Common Vision	Total Internal Social Capital
**Special nurse**	No (REF)						
	Yes	0.047***	0.025***	0.203*	1.098	0.800***	0.221**
**Volunteer**	No (REF)						
	Yes	1.807	3.162	11.721*	2.201	5.485	2.975
**Types of services**	≤ 3(REF)						
	4–6	8.529	57.953*	52.422*	4.108	3.603	1.319
	≥ 7	0.639	6.650	3.297	2.536	0.938	0.542
**Management employees**	≤ 3(REF)						
	4–6	-0.678	0.412	1.655	1.939	0.375	1.351
	≥ 7	0.245	0.556	-0.176	0.542	0.499	0.604
**full-time employees**	≤ 15(REF)						
	16–30	6.408	24.247**	4.308	1.810	8.672*	5.730*
	≥ 31	0.282	1.799	4.871	2.621	2.847	1.032
**constant**		8.237	0.719	0.236	0.263	3.170	1.741

^*^p < 0.05

^**^p<0.01

^***^p<0.001

## Discussion

The increase in elderly-caring demand and demand diversification has become a trend. As an important supplement to the pension model, ESOs have gradually moved towards a supporting position. There is now a general consensus that internal social capital has a positive and significant impact on performance [15]. The mechanism for improving the internal social capital is not clear, and there is a lack of scientific quantitative research. In this work, we conduct a quantitative analysis of the data collected, and the results clearly support the view that special care, volunteer, types of services and full-time employees are important for improving internal social capital and its five dimensions of derivation. But our study did not find a link between the number of Management employees and internal social capital.

### Special care and internal social capital

A positive correlation between specialized nurse and internal social capital was found. This interesting revelation to some extent provides explanation for previous studies which have observed that special care for reduced probability of negative events for older adults in nursing homes, such as physical injury (falls), malnutrition, constipation and incontinence, and chronic diseases. The results of our study proved the correctness of explaining the correlation from the perspective of internal social capital [24, 25]

ESOs which provide special care have higher requirements for staff than others. Special care is required to obtain relevant qualification certificates or receive professional training regularly. This perhaps explains the greater probability for quality care. Nevertheless, Jacob has argued in his research that there is a positive correlation between internal social capital within organizations and the quality of care provided by individual care workers, and that increasing manpower or the proportion of trained staff is not in itself a key factor [26]. Inspite of this, special care may play a certain role in improving social support. It is important to emphasize the fact that in ESOs who provide special care, due to the poor self-care ability of older adults, the proportion of caregivers is higher, which can bring spiritual and material support to older adults. Obviously, the provision of special care services is not a simple matter, he encouraged employees to continue to strengthen exchanges between, share experience, so as to better complete the work [27].

### Volunteer and internal social capital

Our research suggests a connection between volunteer staff and internal social capital, and having volunteers often accompanies higher total scores of internal social capital. Dawn A. Marcus [28] argues in his paper that more than half of the volunteers will establish private contact with clients, which will also enhance the organization 's social support for older adults [28–31]. In a qualitative study based on the SPIDER framework, it was shown that community health workers and volunteers can use their own social trust and social resources to do their work better. This was mainly attributed to the foreignness and sense of mission of volunteers. As ' outsiders ', volunteers can bring resources different from those within the organization, and older adults are more willing to share with them. Their participation has greatly enhanced the social support of the organization, and community support has an important impact on the long-term success of the organization [31].

### Types of services and internal social capital

Compared with organizations with ≤ 3 number of services, our research had shown that social norms and social capital score dimensions is high for institutions with 4–6 services. When ESOs provide 4–6 kinds of services, they will be more focused on implementing this service and specific operational specifications can be formulated for services within the organization. At the same time, due to the relatively small number of services provided, employees can focus on improving service quality and comply with organizational requirements. It has be identified that A previous survey we conducted in Anhui Province showed that the number of services provided by ESOs is also one of the determinants of the choice of nursing home for older adults [32]. This highlights the need for parity with respect to the number of services offered and the internal social capital of ESOs. A study based on China 's national conditions shows that ESOs need to balance the relationship between service quality and service type in their development, and gradually develop towards specialization, rather than blindly increasing full-time employees and service types [33]. This was partly consistent with our research results.

### Full-time employees and internal social capital

Institutions with 16–30 full-time employees were more likely to get higher scores on social norm, shared language and shared vision and total social capital compared to ESOs with less than 15 full-time employees. This finding contradicts previous studies have shown that there was no obvious relationship between full-time employees and social capital, while this paper revealed the hidden relationship between full-time employees and social capital [34]. This can be interpreted that when the number of employees in the ESOs is small, to accomplish tasks well, more communication and interaction between members are needed. While the organization exceeds a certain size, the more employees there are, the more detailed the division of labor will be, which reduces the opportunities for communication among the members of the organization.

The number of services and the number of full-time employees were two indicators reflecting the size of the company. In the field of enterprise efficiency, Dhawan has calculated and compared the average profit margins of small, medium and large companies with panel data of US listed companies from 1970 to 1989, and found that small and medium enterprises are more efficient [35]. And similar research results in the field of pension. A study based on China 's national conditions also shows that ESOs need to balance the relationship between service quality and service type in their development, and gradually develop towards specialization, rather than blindly increasing full-time employees and service types [33].

The core elements of internal social capital, including social networks, trust, support, norms, common language, and shared vision, are lacking in ESOs in the healthy aging service field. At the same time, internal social capital has the function of helping organizations acquire resources, improving organizational capabilities, and promoting organizational performance. Our study provides a new perspective and approach to explore strategies for ESOs to participate in healthy aging services. It also serves as an effective exploration in comprehensively addressing the various challenges faced by ESOs. By nurturing and enriching the stock of internal social capital in ESOs, promoting active and effective involvement of governments, multiple sectors, and ESOs in healthy aging services, it contributes to the positive interaction among governments, multiple sectors, and ESOs in achieving healthy aging objectives.

## Limitations

Although this article has some innovations and some new discoveries, it is still insufficient. This survey was carried out in Chongqing, China, and the conclusions drawn are only applicable to provide reference for the development of ESOs in Chongqing. The study involved a small sample size of 80 ESOs due to challenges related the COVID-19 Epidemic Control Measures; thus, limiting the generalizability of the findings. We suggest further studies involving a large sample size to explore the important role of the four factors of special care, volunteer, the types of services and full-time employees for the development of ESO services industry.

## Conclusion

In summary, organizations with different basic conditions have different amounts of social capital. Factors such as the presence of special care and volunteer staff, a certain number of full-time staff as well as types of services rendered are positively associated with the internal social capital of ESOs. From the five factors discussed in this study, we present the following recommendations: Firstly, adapting to the changes in national pension needs and systematically developing towards special care institutions can effectively improve the service capacity of institutions. Gradually, explore the introduction of registered nurses into ESOs. Secondly, establishing a good volunteer training relationship with schools or communities to engender volunteerism for ESOs. Furthermore, the medium quantity of service types would centralize institutional resources and would also be more attractive to older persons. Finally, we submit that attention should be given to the number of agency staff in view of its manifestation in communication and overall effect on social capital.

### Supplementary Information

**Additional file 1.**

## Data Availability

The datasets analysed during the current study are available from the corresponding author on reasonable request.

## Abbreviations

M(IQR): Median(interquartile range)
REF: Reference
