# Supplementary material for: Factors related to the internal social capital of elderly-caring social organizations: a cross-sectional study in Chongqing, China
Source: BMC Health Serv Res. 2023 Aug 23;23:896. doi: 10.1186/s12913-023-09912-8 (PMC10463601; doi:10.1186/s12913-023-09912-8)
Supplement: Supplementary file 1 — Additional file 1. [file 12913_2023_9912_MOESM1_ESM.docx]

**Questionnaire for superintendents of social organizations in the field of elderly care services**

**Part A Basic information of** **the ESOs**

| **A** **Basic information** |
| --- |
| **A1 Per capita floor area(m^2^):**①≦50; ②﹥50 |
| **A2 Founding time(years) :**①≤2; ②3-8; ③≥9 |
| **A3 Full-time staff:**①≤15; ②16-30; ③≥31 |
| **A4 Management employees:**①≤3; ②4-6; ③≥7 |
| **A5 Owning Volunteer Staff:**①Yes; ②No |
| **A6 Types of Registration:**①1; ②﹥1 |
| **A7 Operational pattern:**①Public construction and operation; ②Public construction and private operation; ③Private construction and operation |
| **A8** **Types of services:** ①≤3; ②4-6; ③≥7 |
| **Service object type** |
| **A9 Self-care:** ①Yes; ②No |
| **A10 Total care:** ①Yes; ②No |
| **A11** **Specialized care:** ①Yes; ②No |
| **A12** **Home care service for the elder:**①Yes; ②No |
|  |

**Part B Internal Social capital of the ESOs**

| **B1 Social network** | | | | | |
| --- | --- | --- | --- | --- | --- |
| **Entries:** | ①0 | ②1-5 | ③6-10 | ④11-15 | ⑤16 and above |
| **B1.1** Frequent personal contacts between superintendents ( e.g. informal gatherings, networking, etc., the same below) |  |  |  |  |  |
| **B1.2** Frequent personal contacts between superintendents and general members |  |  |  |  |  |
| **B1.3** Frequent private contacts between general members of the organization |  |  |  |  |  |
| **B1.4** Frequent working contacts between superintendents ( e.g. regular / irregular meetings, daily working exchanges, discussions, etc., the same below ) |  |  |  |  |  |
| **G1.5** High level of interaction between superintendents and general members |  |  |  |  |  |
| **G1.6** Frequent working contacts between general members of the organization |  |  |  |  |  |
| B2 **Social trust** | | | | | |
| **Entries:** | ①0 | ②1-5 | ③6-10 | ④11-15 | ⑤16 and above |
| **B2.1 Members of the organization keep their promises to each other and act in accordance with their words** |  |  |  |  |  |
| **B2.2 Members of the organization are loyal and accountable to the organization** |  |  |  |  |  |
| **B2.3 Members believe that they can actively cooperate with each other** |  |  |  |  |  |
| **B2.4 Members believe that core members can promote the development and progress of the organization** |  |  |  |  |  |
| **B2.5 When members of the organization have temporary problems, they are assured that the task to other members to complete** |  |  |  |  |  |
| **B3 Social support** | | | | | |
| **Entries:** | ①0 | ②1-5 | ③6-10 | ④11-15 | ⑤16 and above |
| **B3.1 The organization provides members with training or retraining opportunities** |  |  |  |  |  |
| **B3.2 When organization members encounter difficulties, the organization can give timely material support** |  |  |  |  |  |
| **B3.3 When organization members encounter difficulties, the organization can give timely spiritual support** |  |  |  |  |  |
| **B3.4 Timely information support within the organization when organization members encounter difficulties** |  |  |  |  |  |
| **B3.5 organization members encounter difficulties, the organization can give timely technical support** |  |  |  |  |  |
| **B4 Social norm** | | | | | |
| **Entries:** | ①0 | ②1-5 | ③6-10 | ④11-15 | ⑤16 and above |
| **B4.1 The Organization has more comprehensive daily management practices** |  |  |  |  |  |
| **B4.2 The Organization has more comprehensive service practices / standards** |  |  |  |  |  |
| **B4.3 Comprehensive performance appraisal system and reward and punishment system in the Organization** |  |  |  |  |  |
| **B4.4 Organization members are familiar with and comply with work processes and practices** |  |  |  |  |  |
| **B4.5 Organization members have a set of default codes of conduct and guidelines for their work** |  |  |  |  |  |
| **B5** **Common Language and Common Vision** | | | | | |
| **Entries:** | ①0 | ②1-5 | ③6-10 | ④11-15 | ⑤16 and above |
| **B5.1 Organizational members are aware of the expertise and procedures involved in their daily work** |  |  |  |  |  |
| **B5.2 Organization members understand the professional symbols and terminology used in their daily work** |  |  |  |  |  |
| **B5.3 Smooth communication and exchange between members of the organization** |  |  |  |  |  |
| **B5.4 members agree with the organization 's phased work objectives** |  |  |  |  |  |
| **B5.5 Members agree with the strategic direction of the organization** |  |  |  |  |  |

Investigator：

Quality controller：

Investigation time：
